# Pamphlets Received

**Published:** 1886-02-20

**Authors:** 


### BOOKS AND PAMPHLETS RECEIVED.

*A Text-book on Hygiene;* a Comprehensive Treatise on the Principles and Practice of Preventive Medicine from an American standpoint. By George H. Dohe, M. D., Professor of Hygiene, College of Physicians and Surgeons, Baltimore; Member of the American Health Association; of the American Dermatological Association; of the Medical and Chirurgical Faculty of Maryland; Corresponding Member of the New Orleans Academy of Science, etc. Baltimore: Thomas H. Evans. 1885.

A work of 314 large octavo pages, in which the department of Hygiene is treated in twenty chapters, embracing the following general heads, viz: Air, Water, Food, Removal of Sewage, Construction of Habitations, Construction of Hospitals, Schools; Industrial Hygiene; Military and Camp Hygiene; Marine Hygiene; Prison Hygiene; Exercise and Training; Baths and Bathing; Clothing; Disposal of the Dead; Germ Theory of Disease; Contagion and Infection; History of Epidemic Diseases; Antiseptics; Quarantine, and Vital Statistics.

*Practical Treatise on Massage;* its History, Mode of Application and Effects; Indications and Contraindications; with Results in over fourteen hundred cases. By Douglas Graham, M. D., Fellow of the Massachusetts Medical Society. New York: William Wood & Co. 1885. 280 octavo pages.

The author of the above interesting work has, we think, well succeeded in his professed object—that is, to resuscitate the most valuable of the many articles on the subject of Massage, as scattered throughout medical literature, and to give the results of his own extensive experience as illustrated by actual cases. The subject is one which is but little understood, and which ought to be studied by medical men.

*Treatise on Nervous Diseases,* their Symptoms and Treatment; a Text-book for Students and Practitioners. By Samuel G. Webber, M. D., Clinical Instructor in Nervous Diseases, Howard Medical School; Visiting Physician for Diseases of the Nervous System at the Boston City Hospital; Member of the Massachusetts Medical Society; Member of the American Neurological Association, etc. New York: D. Appleton & Co. 1885.

The above is an ably written work of 415 pages, octavo, and is designed especially for students and general practitioners. After the general introduction on the subject of Testing Sensation, Methods of Treating Motion, Reflexes, Nutrition, etc., the succeeding chapters treat principally of brain diseases; diseases of membranes; change in blood supply; hemorrhage; occlusion of cerebral arteries; diseases of spinal cord; diseases of peripheral and sympathetic nerves, spasm, etc.; virtigo, chorea, paralysis agitans, epilepsy, hysteria, neurasthenia, tentanus, toxic neurosis, syphilis, etc. It is a work of marked ability and merit.

